# EMBRACE-ing a Second Wave of Trauma-Informed Research and Practice in Schools: An Embodied and Relational Approach

**DOI:** 10.1007/s40653-026-00912-4

**Published:** 2026-06-04

**Authors:** Catriona O’Toole

**Affiliations:** Department of Education, Maynooth University, Maynooth, Co Kildare Ireland

**Keywords:** Trauma-informed, Schools, Education, Equity, Social justice, Relational, Embodied

## Abstract

Trauma-informed education has gained significant traction in recent decades, yet prevailing models often adopt an individualised, decontextualised approach that fails to address the systemic and relational nature of trauma. This article calls for a “second wave” of trauma-informed research and practice in schools, underpinned by a relational and embodied ontology. It introduces the EMBRACE Framework (EMBodied and RelAtional trauma-informed practice within sChool Ecosystems), a model designed to guide the enactment of trauma-informed approaches at both the individual practice and whole-school levels. Informed by a state-of-the-art review, ongoing research and collaborative engagement, the framework adopts a compassion-centred and reflective approach, and emphasises systemic change within four domains of school life: policies and practices, culture and ethos, curriculum and pedagogy, and relationships and partnerships. Additionally, it highlights the need for equity-driven and rights-based perspectives to address the socio-cultural and political dimensions of trauma. EMBRACE offers a generative and flexible model to support future progressive work in trauma-informed education. Its distinctive value lies in how it synthesises and organises core elements into a coherent and unifying whole, rather than presenting them as stand-alone or parallel aspects of best practice. In doing so, the framework supports schools in cultivating the conditions for hope, healing, and meaningful systemic change, an endeavour that is more urgent than ever.

Over the past two decades, trauma-informed practices have emerged as a transformative approach in educational settings, aiming to support children impacted by trauma while fostering inclusive and positive school climates. This movement was initially inspired by the landmark Adverse Childhood Experiences (ACE) study, which illuminated the pervasive impact of childhood adversity on long-term health and development (Felitti, et al., 1998). However, the translation of these findings into schools, referred to as the “first wave” of trauma-informed practices (Chafouleas, et al., 2021), has faced criticism limiting its potential to drive systemic change.

The limitations of this first wave are evident in its tendency to conceptualise trauma primarily as a set of behavioural symptoms or neurobiological responses, thereby overlooking the deeply embodied and relational nature of traumatic experience (O’Toole, 2022; Petrone & Stanton, 2021). This reductionist orientation has given rise to interventions that focus largely on individual skill-building or behaviour modification, while neglecting the wider social, cultural, and environmental contexts in which trauma is produced and sustained (Becker-Blease, 2017). Insufficient attention has been given to structural inequalities and other social determinants of adversity (Kelly-Irving & Delpierre, 2019). This has contributed to deficit-based framings that position trauma-affected students as problems to be managed, rather than recognising their strengths, agency, and capacities for healing and connection (O’Toole, 2022). Such approaches can perpetuate deterministic and fatalistic narratives, further stigmatising those already marginalised. Indeed, several authors, including Robert Anda, one of the original architects of the ACE study, have raised concerns about the misuse and misapplication of ACE research (Anda, et al., 2020; Finkelhor, 2018), particularly the inappropriate deployment of the ACE questionnaire as an individual screening tool, contrary to its intended purpose.

Part of the problem was that early attempts to translate ACE research into educational practice were shaped largely by narrow disciplinary perspectives, particularly from medicine and mental health (Thomas, et al., 2019). These silos produced overly simplified understandings of trauma and of the kinds of responses required in complex educational settings. No single field can fully account for the challenges faced by trauma-affected students; neurophysiology, psychology, education, social work, youth justice, and rights-based approaches each illuminate different aspects of how trauma is experienced in schools. Crucially, academic knowledge must also be integrated with the lived expertise of practitioners, students, and families. Without such cross-disciplinary engagement, first-wave approaches, despite good intentions, led to responses that were narrow, individualised, and insufficiently attuned to the multifaceted and contextual nature of trauma. Thomas and colleagues (2019) further note that education has played a marginal role, even though schools are central sites of trauma identification and response. Teachers are often positioned merely as referral agents or implementers, rather than as professionals whose pedagogical practice is integral to the system of care. This imbalance constrains understanding of educators’ work and of what trauma-informed teaching should entail.

Whilst first-wave trauma-informed practices in schools show promise, with reported benefits across multiple student domains - academic, emotional, and behavioural (Blitz et al., 2020; Crosby, 2016; Dorado et al., 2016; Stokes & Turnbull, 2016), and improvements in teachers’ quality of life, knowledge, skills, and confidence (MacLochlainn et al., 2022; Swift, 2023); most evaluations have focused on individual or group-based interventions targeting trauma-affected individuals. There is a dearth of research exploring the implementation and effectiveness of whole-school and multi-tiered approaches (Avery et al., 2021; Berger, 2019; Maynard, 2019). This is despite growing consensus that the core principles and values of trauma-informed practice must be fully integrated into the fabric of school life (Treisman, 2017). Rather than being compartmentalised into discrete elements of practice, trauma-informed approaches require a fundamental shift in how educators think about and deliver education (Avery et al., 2022). Researchers in the field have identified that future work in this area needs to address intersecting issues such as human rights, cultural trauma, equity, and schools as environments of healing and hope (Avery et al., 2022; Chafouleas et al., 2021). They have also highlighted the necessity of system-level change, which requires adaptations to both individual practice and the school as a whole (Avery et al., 2022; Berger, 2019). A shared conceptual framework that attends to the complexities of school environments is also required (Thomas, et al., 2019).

## Towards a Second Wave of Trauma-Informed Practice in Education

To move beyond the limitations outlined above, this article proposes a second wave of trauma-informed research and practice in education, one that attends to equity, inclusion and children’s rights, and considers both individual and systems-level practices. Rather than aligning itself with any single disciplinary tradition, a second wave calls for a deeper ontological grounding to support transdisciplinary thinking. Drawing on contemporary understandings of trauma and healing, this paper advances a *relational and embodied ontology*: a philosophical stance that recognises that human experience, including experiences of trauma, cannot be understood apart from the living body or from the networks of relationships, environments, histories, and social structures in which each person is embedded (e.g., Barad, 2007; Merleau-Ponty, 1962; Varela et al., 2017).

From this perspective, human beings are not isolated, self-contained individuals but emerge through dynamic relations, interactions, and embodied engagements with others and their environments. Perception, cognition, and emotion are therefore not purely internal processes; they are shaped by bodily states and by the social and material contexts through which experience unfolds (Varela et al., 2017). Childhood trauma illustrates this dynamic (O’Toole & Simovska, 2022). When a child experiences overwhelming threat or adversity, fear, shame, anger, and alienation are registered in the body. These embodied traces shape later perceptions and actions, even when the child lacks a coherent narrative through which the experience can be articulated (van der Kolk, 2014). As Merleau-Ponty’s notion of the *interworld* suggests, thoughts, feelings, and actions arise through an ongoing interplay between body, environment, and social life. By the same logic, healing also unfolds through relational and environmental entanglements: supportive relationships, attuned contexts, and new embodied experiences gradually reconfigure patterns of perception and action, restoring a sense of safety and opening new possibilities for being and acting in the world.

Fundamentally then, a relational ontology foregrounds our fundamental interdependence with others and with the material world, while an embodied ontology highlights that trauma is lived through the body and shapes how individuals inhabit and engage with their worlds. Together, they offer a robust foundation for a second wave of trauma-informed educational practice. This ontological shift is crucial for several reasons. It allows us to better understand trauma as a lived experience that encompasses both personal and collective dimensions, shaped by cultural, historical, and sociopolitical forces. It also highlights the importance of addressing not only individual healing but also the transformation of the relational and institutional contexts that may perpetuate trauma.

## Introducing the EMBRACE Framework

**Fig. 1:**
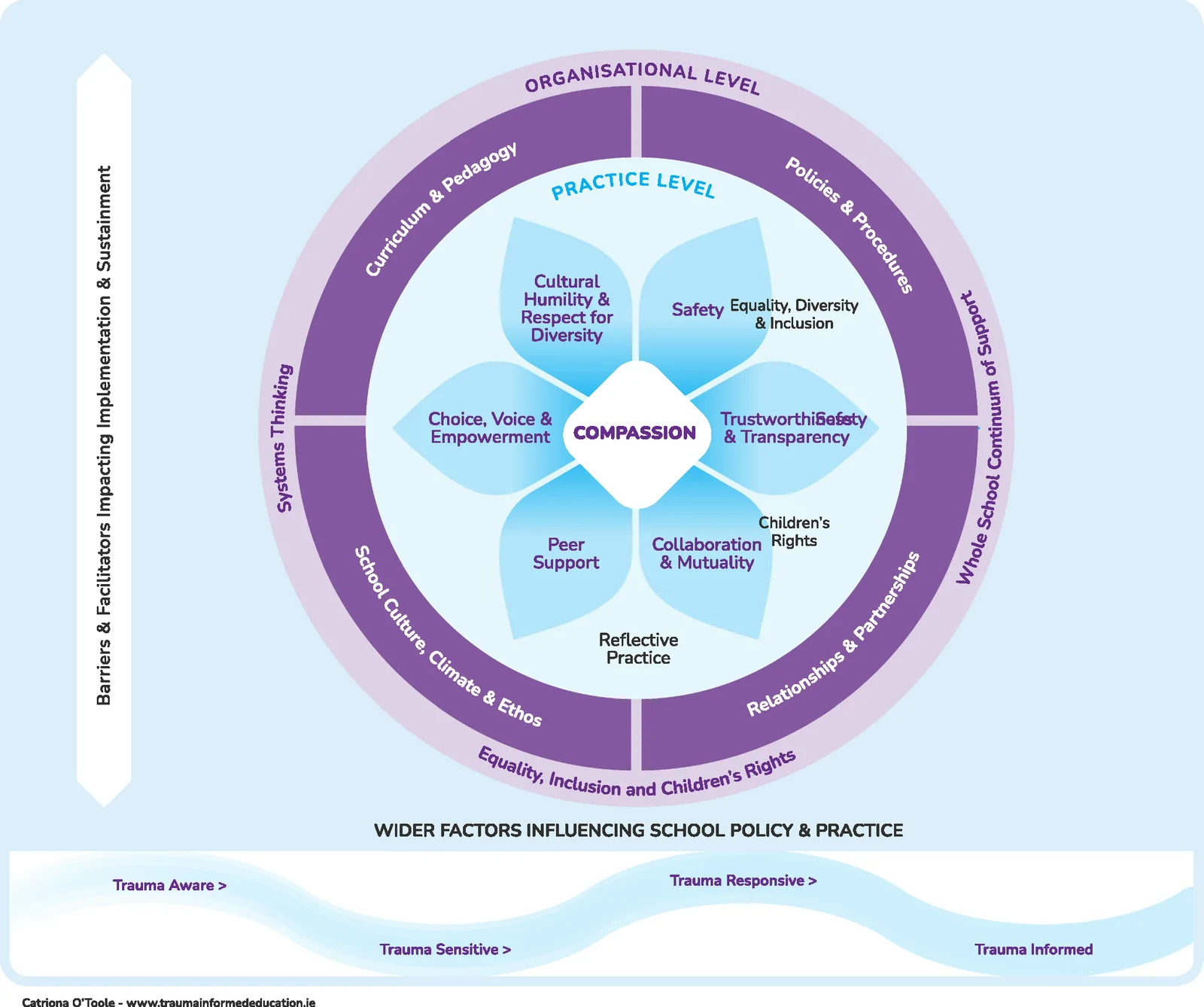
The EMBRACE Framework: A relational and embodied model for trauma-informed practice in schools, integrating individual practice and systemic approaches to foster healing, connection, and equity

Building on these foundations, this paper introduces the EMBRACE framework (EMBodied and RelAtional trauma-informed practice within sChool Ecosystems), a compassion-centred model designed to guide trauma-informed initiatives in schools. The framework was developed through the application of state-of-the-art review methodology (Grant & Booth, 2009) to map and assess the existing knowledge base on trauma-informed practice in education. This was complemented by a sustained programme of research conducted by myself, my team and collaborators, and enriched by practice-based insights generated through ongoing collaborations and knowledge-exchange activities with educators and other practitioners across diverse settings. Following this conceptual work, the key components of the framework were identified and initially sketched, after which a graphic designer developed the visual representation presented in Fig. 1. Although grounded in rigorous scholarship, the EMBRACE framework is advanced with a recognition of the contextual and positional influences that shape its development. It is not a definitive or prescriptive model, but as a generative starting point from which further dialogue, refinement, and collaborative evolution can emerge. It is intended to support continued progress in trauma-informed educational practice while remaining open to adaptation, critique, and contextual reinterpretation.

As illustrated in Fig. 1, trauma-informed practice is symbolised as a flower, with six core principles represented as petals: safety, trustworthiness and transparency, collaboration and mutuality, peer support, voice, choice and empowerment, and cultural humility and respect for diversity (Harris & Fallot, 2001; SAMHSA, 2014). Like a flower, beautiful yet fragile, trauma-informed practice requires the right conditions to grow and flourish. These conditions, the barriers and facilitators of successful implementation, are denoted on the left-hand side of the Framework. The Framework is represented by concentric circles. The inner circle represents the practice-level, emphasising the changes that individual practitioners can make to embed trauma-informed ways of working into their own individual practice. The outer circle represents organisational-level change, which is about infusing trauma-informed principles within the school as a whole. The path at the base of the Framework underscores that trauma-informed practice is an ongoing, evolving process; a journey that progresses from trauma awareness to sensitivity, responsiveness, and ultimately trauma-informed (Carter & Blanch, 2019). As with any journey, the road may not always be straightforward; there may be twists and turns along the way. Yet it is a journey that is both meaningful and necessary, perhaps most especially in the context of current geopolitical upheavals. In the following sections a rationale for key elements of the framework is discussed, starting with compassion which is positioned at the heart of the framework.

### The EMBRACE Framework at Individual Practice Level

#### A Compassion-Centred Approach

The EMBRACE framework adopts a compassion-centred approach underscoring the need for a paradigm shift from isolated interventions to a holistic approach that integrates relational and embodied principles. Compassion can be defined as the recognition of suffering in oneself and others, accompanied by a genuine desire to alleviate it (Gilbert & Choden, 2013; Strauss et al., 2016). As this definition suggests, compassion is a multidimensional concept, encompassing affective (empathy, kindness), cognitive (awareness, non-judgment), and motivational (the desire to alleviate suffering) dimensions. Khoury (2019) further emphasises the embodied and relational nature of compassion, aligning with the ontological stance discussed above. Compassion is experienced and expressed through the body via touch, tone of voice, posture, movement, and facial expression, whilst also deeply embedded in interpersonal and social contexts. It is not simply a feeling or an action but a transformative *way of being* with the potential to reshape relationships within the school community (Avery et al., 2022; Senge et al., 2019).

Physiological studies provide compelling empirical justification for foregrounding compassion: they show that compassion activates neural systems associated with safety and social affiliation, which in turn enhance emotional regulation and reduce stress (Engen & Singer, 2015; Kok, et al., 2013; Porges, 2017; Svendsen et al. 2016). In educational settings, compassion-based interventions have been shown to lower physiological stress in adolescents and reduce teacher burnout, creating optimal environments for learning and growth (Akpan & Saunders, 2017; Dave, et al., 2020; Flook, et al., 2013; Jennings, 2015; Matos, et al., 2022).

Compassion can be directed both inward towards the self (self-compassion) and outward, towards others. Teacher self-compassion and wellbeing are crucial in the context of trauma-informed practice (O’Toole & Dobutowitsch, 2023). Working with students who have trauma histories can bring up strong emotional reactions that can influence teachers’ practice. Without a means of adaptively processing their experiences and reactions, teachers often mask their feelings (Chang, 2009) increasing their risk of compassion fatigue; a state of emotional and physical exhaustion marked by reduced empathy and burnout (Figley, 2002; Keller et al., 2014; Stamm, 2012). In contrast, compassion satisfaction is the positive emotional state gained from helping others, which enhances resilience and job satisfaction (Stamm, 2012). A compassion-centred approach aims to mitigate compassion fatigue while promoting compassion satisfaction, ensuring educators are supported and empowered to sustain their vital roles. This approach is not intended to place the responsibility solely on individuals to prevent compassion fatigue and maintain compassion satisfaction (Edwards & Giussios, 2021). Instead, it calls for systemic structures to support practitioners’ well-being and compassion.

Building on these empirical and theoretical perspectives, the EMBRACE Framework endorses a compassionate-centred approach, as a practical pathway for practitioners interested in the changes they can make to their own individual practice. It also recognises that these individual-level practices can have ripple effects that ultimately transform educational communities into spaces of healing and growth. The EMBRACE Framework thus considers compassion as a prerequisite for the meaningful and authentic enactment of the six core trauma-informed principles (safety, trustworthiness and transparency, collaboration and mutuality, peer support, voice, choice and agency, and cultural humility and respect for diversity). These six principles of trauma informed practice originally proposed by Harris and Fallot (2001) and advanced by SAMSHA (2014) have been described in detail elsewhere. An overview of these principles is provided in Table 1, along with concrete examples of how these principles may manifest in day-to-day practice.

**Table 1:** Trauma-informed principles with illustrative examples from school practice

Principle	Description	Examples
Safety	Creating environments—both physical and emotional—where individuals feel secure, valued, and free from harm or re-traumatisation.	• Soft lighting & furnishings; welcoming signage; plants/flowers • Students’ artwork visible • Create predictable routines • Respond promptly to threats, ridicule, displays of power • Greet students/parents at the gate every morning • “Welcome!”, “How are you!”, “Great to see you!”
Trustworthiness & Transparency	Building integrity and reliability through clear communication, consistency, and honesty, reducing power imbalances to create predictable and dependable relationships.	• Say what you mean & mean what you say! • Open, sincere communication • Practice what you preach • Ensure frequent check-ins • Regular circle time • Admit mistakes • “I’ll always be honest with you” • “I got that wrong, I’m sorry…”
Collaboration & Mutuality	Recognising the power of shared decision-making, valuing all voices, and building relationships based on respect and partnership rather than hierarchy.	• Nothing about us, without us! • Co-create classroom agreements with students rather than imposing rules. • Avoid surprises – let students know what to expect • Use restorative practices instead of punitive discipline • Avoid minimising or dismissing student concerns • “Let’s figure this out together”
Peer Support	Leveraging the strength of shared experiences to promote healing, connection, and a sense of belonging.	• Promote tolerance and respect • Foster friendships • Ensure role models within community • Provide buddy programmes • Offer breakfast club • Offer variety of extra-curricular activities • “You are not alone”
Voice, Choice & Empowerment	Honouring individuals’ autonomy, providing opportunities for meaningful participation, and validating personal experiences to restore a sense of control.	• Ensure school student council with diverse representation • Ensure students have a say in classroom decisions • Offer choice boards • Validate students’ emotions and perspectives, ensuring they feel heard • “You always have a choice – let’s find an option that works for you”
Cultural Humility & Respect for diversity	Acknowledging and addressing power imbalances, valuing different perspectives, and committing to continuous learning to create inclusive and affirming spaces.	• Recognise own biases & blind spots • Check assumptions • Rember students’ names and pronouns • Demonstrate humility & openness to learning • “Thank you for sharing your experience” • “If I say something that doesn’t sit right with you, please let me know so I can address it, we can learn together”

#### Reflective Practice

Reflective practice is a crucial yet often overlooked component of the trauma-informed practice literature. Teachers’ beliefs, values, and assumptions - both implicit and explicit - shape their professional judgments and actions (Fives & Gill, 2015; Johannessen, 2024). Widely regarded as a hallmark of professionalism, reflective practice involves a deliberate and structured process of open-minded inquiry into one’s beliefs and the challenges encountered in the classroom (Day et al., 2022; Postholm, 2008; Schön, 1991).

Critically reflective practice, or ‘praxis’, the ongoing cycle of learning, reflection, and action through which people deepen their awareness and embody their understanding in practice (Freire, 1970), helps teachers recognise how their beliefs and actions are shaped by broader socio-political systems. It can help uncover implicit biases that perpetuate inequalities over time, revealing disparities in how different groups of students are supported, disciplined, or encouraged. This in turn draws attention to how biases uphold, reproduce, or reconstitute oppression and privilege (Roche, et al., 2020). Reflective praxis therefore supports teachers in becoming more effective agents of change by fostering a deep understanding of how their actions impact the communities they serve. It can also suggest possibilities for trauma-informed work in schools to be connected to wider projects for social change (Ginwright, 2015; O’Toole 2022).

Reflective practice also supports the development of intra- and inter-personal competencies necessary for providing genuine compassion and support (Brunzell et al., 2016; Perry, 2009). In the context of trauma-informed care, it is important that teachers develop self-awareness, recognising their own triggers and emotional reactions, navigating relationship rupture and repair, and using the quality of their relationships to foster the co-regulation of trauma-affected students (Treisman, 2017). They also need to cultivate the ability to attune to the inner experiences of children by observing subtle cues such as gestures, demeanour, expressions, and body language (Aguilar-Raab, 2020). From an embodied and relational ontological perspective, reflective practice means attending to all the above-mentioned dimensions - personal, interpersonal, and political.

Various models of reflective practice have been proposed, ranging from solitary reflection (e.g., journalling, expressive writing) to collaborative approaches such as communities of practice (Wenger, 2011), reflective dialogues (Dogan, et al., 2018), and reflective supervision (Lawerence, 2020), as well as more recent technology supported approaches, such as Video Enhanced Reflective Practice (Birbeck, et al., 2015). Research supports the use of reflective practice in the context of trauma and healing (Loomis et al., 2024). For instance, Ginwright (2015) engaged teachers in a *critical inquiry group* focused on healing unconscious racial bias. Southall and colleagues (2023) found that *reflective circles* supported a ‘paradigm shift’ from reactive responses to student behaviour disruptions, toward reflective and empathetic understandings of the trauma behind their student’s behaviours. Involving teachers in the co-design of reflective inquiry methods, may be particularly beneficial (Potvin et al., 2023).

These examples point to the importance of valuing subjective experience as a foundation for knowledge, using critical reflection to examine underlying values, assumptions, biases, and actions (Roche et al., 2020). As trauma-informed research and practice continue to evolve, a second wave must adopt a multi-level approach to reflective practice; one that attends not only to individual thoughts, emotions, and experiences but also to the broader socio-political forces that shape them.

### The EMBRACE Framework at Organisational level

#### A Whole-School Systems Thinking

While research on trauma‑informed practice has expanded rapidly, efforts to scale this work to systemic and whole‑school approaches remain relatively nascent. The outer circles of the EMBRACE Framework foreground organisational and systems‑level change, recognising schools as complex, dynamic social systems shaped by distinct cultures, norms, and ethos. Adopting a systems perspective enables students’ needs to be addressed holistically and supports the coordination, sustainability, and contextual responsiveness of trauma‑informed initiatives within each school. Drawing on Jackson’s Critical Systems Thinking (Jackson, 2019), EMBRACE takes an explicitly integrative and reflexive stance, emphasising the importance of boundary critique, plural perspectives, and ongoing reflection on whose needs, voices, and interests are prioritised. This lens cautions against reductionist approaches to practice and is particularly salient for whole‑school trauma‑informed work, where efforts to foster safety, compassion, and connection must also engage with questions of equity, exclusion, and institutional power embedded within school cultures and policies.

One of the hallmarks of a systems approach in schools is a continuum of support model or Multi-Tiered System of Supports (MTSS). This approach acknowledges that children and young people have varying needs that evolve over time. Those facing greater risks or with more complex needs may require specialised, targeted interventions in addition to the general support provided to all students within their class groups. This approach allows schools to respond flexibly to the changing needs of students, adapting support strategies in line with the school’s resources and specific context (NEPS, 2007). MTSS is represented as a three-tiered model of intervention, including Tier 1 for universal support of all students, Tier 2 for intensive secondary support with groups of students; and Tier 3 for tertiary, intensive and individualised intervention for students with significant difficulties (Sugai & Horner, 2006; Weist et al., 2018). However, MTSS is not value-neutral. Without critical reflection, it can function as a normative framework that privileges particular definitions of success and need. It risks pathologising difference and locating responsibility for change within the child rather than the system. A critical systems thinking (CST) lens is therefore essential, as it foregrounds power, voice, and the boundaries through which support is defined and allocated. From a trauma-informed perspective, this enables structural and relational sources of harm to be surfaced, supporting responses that are ethical, reflexive, and contextually responsive. Consistent with second-wave trauma-informed practice, CST shifts the focus away from managing individual symptoms toward adapting systems to reduce harm, expand participation, and foster collective responsibility for wellbeing.

Drawing on research currently underway (e.g. McKernan & O’Toole, 2024), Table 2 offers examples of universal, secondary, and tertiary trauma-informed supports being explored by practitioners in Ireland. The examples listed in the table, simultaneously engage multiple components of the EMBRACE framework. For instance, restorative practice (presented under the universal tier) functions primarily through relational processes; it requires a compassionate-orientation, engages trauma-informed principles of collaboration and mutuality, whilst also contributing to an equitable and inclusive, school culture. This multiplicity reflects the ecological nature of second wave trauma-informed practice and aligns with systems-thinking approaches to school improvement. Deciding on supports or initiatives should follow CST and co-design principles (see below) to help ensure a contextual fit with the school’s strengths, needs and culture, and to support sustainability over time.

**Table 2:** Examples of trauma-informed initiatives mapped onto the three tiers of MTSS/Continuum of support models

Tier	Examples
Universal: support for all	• Providing high-quality Continuing Professional Development for all staff • Establishing a core team to enact change with support and resourcing from school leadership • Ongoing mentoring and support for staff; including time and space for reflective practice • Auditing and redrafting of school policies to align with trauma-informed principles • Prioritising relationships, e.g., greeting children/families at the school gate every morning • Posters and artwork reflect students’ culture and heritage • Establishing predictable routines, clear expectations and structured transitions • Emphasising restorative practices and relational-enhancing discipline rather than punitive consequences. • Providing calming areas, quiet corners, or movement breaks to help students self-regulate. • Incorporating activities guided by the 6Rs of the neurosequential model (Perry, 2008); or adopting PACE principles (Playfulness, Acceptance, Curiosity, Empathy) to help children feel safe (Hughes & Golding, 2024) • Strengthening school-family-community partnerships (Lindstrom, et al., 2024)
Secondary: targeted or small group support	• Targeted accommodations such as adjusted workload, classroom exit card, staggered start time • Mentoring or Check-in, Check-Out programmes (Drevon, et al., 2018: Hackney, et al. 2024) • Setting up a nurture room (Boxall & Lucas, 2010) • School therapy/support dog (Wintermantel, et al., 2023) • Small group social and emotional learning in nature (Mulholland & O’Toole, 2021). • Peer support groups for students with shared experiences (e.g., grief/loss)
Tertiary: Intensive and individualised support	• Individualised support for strengthening teacher-student relationships, intentional one-on-one conversations and trust building strategies like “banking time” (Williford & Pianta, 2020) and “2 × 10” (Wlodkowski, cited in Mendler, 2001) • Intensive student support plans • Offering personal counselling, play therapy • Onward referral to specialist services • Liaising with other support agencies and family to offer wraparound support

#### Four Interrelated Elements of School Practice

Taking a systems approach, the EMBRACE Framework emphasises multilevel and integrated adaptations to all areas of school practice. Four broad areas of school practice have been identified in research and policy literature: (1) curriculum and pedagogy, (2) relationships and partnerships, (3) school culture, climate and ethos, and (4) policies and procedures (Department of Education, 2019, O’Toole & Darlington, 2021). These four areas are represented in the Framework shown in Fig. 1. The following sections summarise available evidence on how trauma-informed practices are being embedded within these areas of practice.

##### Curriculum and Pedagogy

There is a growing body of research that considers ways that teachers can adapt curriculum and pedagogical strategies to better support trauma-affected students and avoid potential re-traumatisation. Trauma-aware pedagogies share common ground with existing pedagogical strategies already linked to effective and inclusive teaching. Indeed, there is interest in integrating trauma-informed practice with the principles of Universal Design for Learning (UDL), as both approaches emphasise flexibility, accessibility, and the creation of supportive learning environments that accommodate the diverse needs of all students (Jenkins, 2024; Salvador, 2022). UDL’s focus on providing multiple means of engagement, representation, and expression aligns well with the trauma-informed principles, particularly the principle, *voice*,* choice and empowerment.*

There is also growing awareness that certain subject areas contain sensitive, challenging and potentially triggering content and activities. For instance, given the public nature of participation and the emphasis on the body in physical education, students may feel acutely self-conscious under the ‘gaze’ of others, especially in highly charged spaces like changing rooms (Quarmby et al., 2022). Similarly, subjects such as history, literature, and global citizenship education often address themes like war, hunger, homelessness, racism, displacement, and death. These topics can be potentially distressing for trauma-affected students particularly when they intersect with their lived experiences (Hancock & Richardson, 2022). Conversely, it is possible that exposure to content reminiscent of their experience serves to validate students’ experiences and foster personal growth (Carello & Butler, 2015).

Given varied student responses to sensitive and potentially unsettling material, navigating curriculum content can be challenging for educators (Harrison, et al., 2018). Dutta and colleagues (2024) caution that in their efforts to be caring practitioners, teachers may inadvertently ‘sanction ignorance’ (Spivak, 1993) by avoiding sensitive topics. This can unintentionally suppress classroom discussions, silence student voices, and limit opportunities for shared learning. Moreover, teachers may not always know which of their students have experienced trauma or how it affects them. For these reasons, EMBRACE emphasises, not the avoidance of sensitive subject matter, nor assumptions about its impact on specific students, but rather a commitment to reflecting on and applying the values and principles of trauma-informed practice across all subject domains. For example, providing advance notice of content and activities reflects the principle of *safety*; offering students choices in how they demonstrate learning supports *voice*,* choice*,* and empowerment*; and providing regular check-ins fosters *trust and trustworthiness*.

Recognising the reality of trauma and its impact, many schools are implementing a range of body-based and relational approaches to support students. Examples include, the neurosequential model (Perry, 2009; Perry & Hambrick, 2008), which integrates rhythmic, patterned activities (such as walking, drumming, and singing) into daily routines to help regulate the body’s stress response system. Mind-body practices like Capacitor (breathwork, fingerholds, visualisation, and centring exercises) provide accessible culturally sensitive healing strategies suitable for classroom use (Crane & Duennes, 2005). The restorative benefits of natural outdoor environments have also been leveraged to reduce stress and enhance student well-being (Mulholland & O’Toole, 2021). In addition, nurture groups offer small, structured settings where students can develop positive relationships with teachers and peers, addressing early relational gaps (Boxall & Lucas, 2010). Relational pedagogy (Morgan, 2015) emphasises trust and connection as fundamental to learning, while creative arts pedagogies can be used thoughtfully to offer a means of emotional expression and communication (Piazzoli et al., 2024). These initiatives highlight some advances in integrating trauma-informed principles into ongoing pedagogical and curricular practices.

Recent research also highlights the alignment between trauma-informed practice and well-established educational approaches. Phillips and colleagues (2022) highlighted that Maria Montessori’s approach to education was trauma-informed in all but name. From the outset of her career, Montessori worked with children who had experienced significant adversity, including children detained in asylums, living in extreme poverty, and left orphaned and homeless by natural disaster. With careful attention to the environment and the integration of calming, regulating activities, along with self-correcting and sensorial materials designed with gradations and sequencing, early Montessori schools gained international recognition as ‘healing schools’ that supported children’s emotional and psychological recovery (Phillips et al., 2025).

##### Policies and Procedures

As previously noted, trauma-informed practice in schools requires a holistic shift in policies and procedures to create environments that engage all trauma-informed principles; that foster safety, trust, collaboration, and empowerment for all students. An important starting place for schools is building knowledge and awareness amongst staff of trauma and its impacts on mind, body and behaviour. This requires high-quality, professional development opportunities for staff to equip them with the knowledge, attitudes and skills to recognise and respond to trauma (Anderson et al., 2015; Koslouski & Chafouleas, 2022). Another crucial aspect of trauma-informed practice is ensuring access to wellbeing resources for staff themselves, helping to sustain their capacity to provide compassionate care, preventing compassion fatigue and burnout. Key components include dedicated time and resources for reflective practice and fostering staff wellbeing, through the provision of professional supervision and counselling or support services (Lawerence, 2020; O’Toole & Dobutowitsch, 2023; Watson et al., 2024).

Schools also need to consider their policies and procedures from a trauma-informed lens, including policies on school behaviour, admissions, attendance, child protection and so on. Trauma-informed practice requires a shift from harsh disciplinary policies (punitive, exclusionary and zero-tolerance approaches), which are known to have negative impacts and to be disproportionately used against marginalised students (Watson et al., 2024). Many schools are shifting from behaviour-focused discipline to relational and restorative approaches, which emphasise repairing harm and rebuilding relationships after conflict, instead of taking a punitive or shame-based approach (Lodi et al., 2022; Vaandering, 2013). Complementing these efforts are anti-bullying strategies, and other initiatives designed to create a school climate that prioritise inclusivity, respect, and the psychological safety of every student. Such policies are often situated in a broader commitment to student mental health, equity and inclusion (Watson, 2024).

Trauma-informed practice (and school wellbeing initiatives more generally) often compete with other demands on schools, such as pressures to achieve academic outcomes and maintain high attendance rates (O’Toole & Simovska, 2022). These priorities can sometimes displace the compassion-centred values integral to trauma-informed approaches. The inherently complex and dynamic nature of school environments underscores the importance of ongoing reflective practice to navigate these competing demands. Strong leadership is also crucial for promoting coherence and fostering ‘joined-up thinking’ across the school community (Dix et al., 2012; O’Reilly et al., 2018). Designating and supporting a ‘trauma-informed champion’ - a strategic lead focused on implementing interventions, influencing staff, and minimising competing priorities - can play a pivotal role in embedding trauma-informed principles effectively (Koury & Green, 2017; Mullin et al., 2024).

##### Relationships and Partnerships

As discussed, the EMBRACE framework is centred on compassionate relationships built around the values of safety, trust, collaboration, mutuality, and respect for each person’s worth and humanity. Within school contexts relationships operate at multiple levels including, student-teacher relationships, peer relationships, staff collegiality, partnerships with parents, families and communities, including local referral services and supports.

Trauma can make relationships difficult or complicated, especially since most childhood trauma occurs in the context of children’s intimate relationships (e.g., abuse, neglect, violence, coercion, attachment disruption; Courtois & Ford, 2009). Children’s early experiences may have taught them that other people are hurtful, unpredictable, or untrustworthy; or that they themselves are unworthy of love, care, and protection. It is only through ongoing, positive, relational experiences that children gradually revise, refine, and re-evaluate negative core beliefs they may have about themselves and others (Treisman, 2017). Strong teacher-student relationships are therefore essential. Navigating these relationships can be tricky as teachers must carefully balance providing challenge to foster growth, while ensuring students feel safe – a dynamic that has been described as “working at the edge” (Price et al., 2018). Too much pressure can lead to withdrawal or confrontation and what constitutes a challenge varies among students (Aultman et al., 2009). Furthermore, given past relational or systemic abuses of power, trauma-affected students may be more sensitive to authority and have a ‘narrow window of tolerance’ for emotional arousal (Jordan, 2008; Siegel, 1999). Misinterpreting student responses (e.g., as wilful defiance) can lead to ruptures in relationships especially if teachers take behaviours personally or react defensively (Kidger et al., 2010).

There are several school-based initiatives that help build trusting relationships and foster connection and belonging (Wilson-Ching & Berger, 2024). Examples include the Check and Connect programme (Anderson et al., 2004), teacher-student mentoring (Banks & Smyth, 2021), peer buddy programmes (Tzani-Pepelasi et al., 2019), breakfast and afterschool clubs (Defeyter et al., 2015). Additionally, pedagogical and curricular initiatives in wellbeing promotion and social-emotional learning reinforce these efforts (O’Toole & Simovska, 2022; Zinsser et al., 2013). All these initiatives centre on attuned, supportive relationships and broadly align with trauma-informed values.

Strengthening relationships with families and community agencies is also vitally important and involves schools engaging in partnerships that acknowledge and respect the lived experiences of families. This can include offering workshops for parents on trauma awareness and providing spaces for families to share their stories and needs (Chafouleas et al., 2016). Building trust with families requires consistent communication and a non-judgmental stance, recognising that families often carry their own trauma histories. Collaborations with community and referral agencies expand the support network for students, providing access to specialised services such as mental health care, housing support, or legal assistance. These partnerships help schools create a holistic, wraparound support system, addressing the multifaceted needs of students and their families in a compassionate and coordinated manner (Haight et al., 2023).

All the above is supported when healthy and supportive relationships are modelled by school staff and supported by organisational structures. Staff relationships also benefit from a trauma-informed culture that emphasises safety, collaboration, and mutual support. As noted earlier, providing time and space for reflective practice, including for instance, regular team meetings, supervision, and spaces for open dialogue, allow staff to process their experiences and share strategies for supporting students, enhancing a sense of collective efficacy and well-being.

##### School Culture, Climate, and Ethos

Strengthening school culture, climate, and ethos from a trauma-informed perspective requires a multifaceted approach that addresses both the physical and emotional environments of the school. Creating safety (a key trauma-informed principle) means designing welcoming physical environment involves creating spaces that are safe, clean, and aesthetically pleasing, which can reduce stress and foster a sense of calm (Souers & Hall, 2016). Incorporating calming colours, natural light, and quiet areas for relaxation can help students and staff feel more at ease. Signage and artwork that celebrate diversity and inclusion further signal that the school values and respects all individuals, enhancing the sense of psychological safety.

Psychological safety (in addition to physical safety) is foundational in a trauma-informed school culture, as it allows students and staff to feel secure enough to take risks, express themselves, and engage fully in the learning process (Sackett, 2017). This can be fostered by establishing clear, consistent expectations and routines, as well as by promoting respectful and empathetic communication throughout the school community. A sense of belonging is cultivated when students feel supported and valued by their teachers and when their unique identities and contributions are recognised (Allen et al., 2018). Culturally responsive teaching practices and the celebration of diverse backgrounds and experiences offer ways to achieve this (Cragg & Kelly, 2018). Creating multiple opportunities for success is another critical component of a positive climate in trauma-informed schools. Recognising and celebrating a wide range of achievements, not just academic success, helps all students experience competence and self-worth (Craig, 2016).

Creating a school culture in which students are genuinely consulted, their perspectives valued, and their input meaningfully acted upon is fundamental in trauma-informed education, aligning with principles of *collaboration and mutuality*, and c*hoice*,* voice and empowerment.* The Lundy Model of child participation (Lundy, 2007) offers a structured approach to promoting student voice through four interrelated elements: Space, Voice, Audience, and Influence. Meaningful student participation requires intentional efforts to dismantle rigid hierarchical structures in schools and replace them with participatory, democratic practices. By shifting from a model in which students are passive recipients of knowledge to one where they are active agents in their educational experience, schools can cultivate environments that promote autonomy, agency, and leadership. In doing so, student voice becomes not only a mechanism for individual empowerment but also a pathway toward more equitable and inclusive educational spaces (Crosby et al., 2023).

#### Equity, Inclusion and Children’s Rights

As the field of trauma-informed education evolves, there is an increasing recognition that research and practice must attend to issues of equity and inclusion (Liasidou, 2024; Walsh & Kelly, 2021). Trauma does not occur in a vacuum; it is deeply rooted in the historical and ongoing social inequities, including poverty, racism, sexism, oppression, and other imbalances of power (O’Toole, 2022). Schools themselves can also be a source of further harm, as evidenced by the experience of many marginalised groups who have faced colonial, racist, classist, sexist, and other oppressive educational policies and practices (Duane et al., 2023: Grant et al., 2022). The systemic racism and oppression that occur in larger society, are often replicated and, at times, amplified in schools, making schools “sites of suffering” for members of those communities (Dumas, 2014; Onipede, 2024).

On one hand, concerns have been raised about the lack of attention paid to issues of power and inequality in current, first-wave trauma-informed practice models (Onipede, et al., 2024). Aligned with a largely outdated medical model, trauma can be viewed in a reductive and individualistic way, failing to acknowledge the systemic factors that contribute to traumatic experiences (O’Toole, 2022; Petrone & Stanton, 2021). Additionally, trauma-informed practices, along with social-emotional learning and behaviour management approaches, have been criticised for their potential to further harm students through deficit-laden narratives of families and communities (Grant et al., 2022). There is also the risk that trauma-informed practice is adopted in the service of white saviourism, enabling dominant groups feel good about themselves (e.g. by helping others), rather than recognising and attending to the root causes of trauma (Goldin et al., 2021).

On the other hand, many oppressed and marginalised groups have found the literature on trauma to be valuable in understanding the harms – individual, collective, and intergenerational – wrought by social injustices and other imbalances of power (Liasidou, 2022; Johnstone & Boyle, 2018). Several authors have adopted trauma (informed) research and scholarship - often in addition to gender, decolonial, and critical race theories - recognising trauma as a constitutive element of intersectional oppression rooted in the conditions and structures of power that conceal and legitimise social inequalities (Liasidou, 2022). For instance, Indigenous authors have described how legacies of colonisation, forced relocation, assimilation policies, and other forms of systemic oppression, lead to widespread intergenerational trauma, which manifests in various ways, including mental health issues, substance abuse, and disrupted family systems (Braveheart & Chase, 2016; Joyce, 2022; Pihama et al. (2017).

These analyses have important implications for the design of second wave trauma-informed practice in schools. They highlight the importance of equitable and inclusive approaches that cater for diverse student groups and address the conditions and structures that exacerbate harm. They emphasise the need for culturally responsive practices, such as honouring Indigenous cultural knowledge and traditional healing practices, reclaiming positive identities for minoritised students, respecting transgender and non-binary identities, as vital components for healing from historical trauma (Braveheart & Chase, 2016). Examples from the literature include Gibson’s (2023) integration of trauma-informed principles with Indigenous knowledges to offer practical, culturally grounded approaches for supporting students in schools in Aotearoa (New Zealand). Similarly, Ginwright’s (2015) work incorporates spirituality, rituals, and culturally relevant practices, while also encouraging forms of social action, such as protests and community engagement, to cultivate hope and empowerment among students.

In addition to a social justice lens, incorporating a rights-based approach aligned with the United Nations Convention on the Rights of the Child (UNCRC, 1989) may provide a more robust foundation for protecting and upholding children’s fundamental rights. All UN member states, except the United States, have ratified the UNCRC and are required to report on their progress (Vaghri et al., 2011; Renteln, 1996). Scotland has taken this further by directly incorporating the UNCRC into domestic law (Scottish Government, 2024), setting a precedent for embedding children’s rights into national policies. Although the language of trauma is not explicitly referenced in the UNCRC, its provisions clearly affirm children’s rights to safety, protection, and recovery. For instance, Article 19 guarantees protection from violence, abuse, and neglect, while Article 39 mandates specialised support for children who have endured such experiences. Article 12 recognises children as experts in their own lives, affirming their right to participate in decisions affecting them (Naughton, Hughes, & Smith, 2007).

Despite its relevance, children’s rights remain underutilised in trauma-informed education (Bargeman et al., 2021). However, some promising initiatives, such as Scotland’s *Compassionate and Connected Classroom* resource (Education Scotland, n.d.), integrate trauma-informed and attachment-based approaches with a rights-based framework. As trauma-informed practice continues to evolve, incorporating a children’s rights perspective will help ensure that school environments prioritise students’ dignity, well-being, and best interests. This is particularly important given that students who have experienced adversity are more vulnerable to coercive disciplinary measures, which can compound their trauma (Onipede et al., 2024; O’Toole, 2022). Embedding children’s rights into school policies and practices can create a more equitable, empowering educational experience, reduce the risk of re-traumatisation, while fostering healing, participation, and holistic development.

A powerful strategy for promoting voice, equality, and meaningful participation is co-designing trauma-informed initiatives with school staff, students, and families. Co-design is an approach that actively involves those with lived experience, helping ensure that trauma-informed practices are not only inclusive but also culturally and contextually relevant. This approach recognises that individuals are experts in their own lives and that a one-size-fits-all model is rarely effective (McKercher, 2020). Also referred to as co-creation or co-production, co-design is increasingly valued in school-based trauma-informed practices for its emphasis on collaboration, shared decision-making, and mutual respect. When initiatives are co-developed with input from all interested parties, they are more likely to be seen as legitimate, equitable, and responsive, ultimately fostering greater trust and leading to better outcomes for students and the wider school community (Ko et al., 2008).

### Considerations for Implementation and Sustainment

#### Barriers and Facilitators on the Journey Toward Trauma-Informed Schools

While the need for a whole-school trauma-informed approach is well established, implementation remains complex and requires careful, deliberate planning, attending to barriers and facilitators (represented on left-hand side of Fig. 1). Barriers identified in literature include limited resources, lack of organisational structures, insufficient leadership support, and weak stakeholder collaboration (Axelsen, 2017; Garcia et al., 2023). Translating trauma-informed knowledge into everyday practice for sustained behavioural change can be particularly difficult (Wittich et al., 2020). A scoping review on the barriers and facilitators identified five key themes necessary in guiding successful trauma-informed implementation: professional development to build staff capacity and commitment; implementation planning to ensure clear strategies and structured processes for sustained practice; leadership support to foster a culture of change; and stakeholder engagement, which involves collaboration with staff, students, and families to enhance adoption (Wassink-de Stigter, et al. (2022). Staff buy-in also emerged as a crucial factor that can either derail or sustain the success of implementation efforts.

Implementation is often a lengthy process (or journey; as represented in the base of the Framework in Fig. 1), requiring schools to navigate various developmental stages. Carter and Blanch (2019) describe a continuum of four stages in the journey toward a systems level change: trauma-aware, trauma-sensitive, trauma-responsive, and trauma-informed. Each stage involves specific tasks, organisational processes, and indicators of progress. Schools may advance at different rates across various domains and may move back and forth between stages over time. Given this complexity, trauma-informed requiring strategic planning and systemic commitment (Wassink-de Stigter et al., 2022). Building on these research findings, the EMBRACE Framework suggests a coordinated effort from all parties to maximise the likelihood of successful and sustained implementation.

#### Wider Factors Influencing School Policy and Practice

The success, quality, and long-term sustainment of trauma-informed practices in schools are influenced by a range of social, economic, and cultural factors that extend beyond the school itself. National and transnational education policies play a key role in shaping school priorities, with organisations such as the Organisation for Economic Co-operation and Development (OECD) and its Programme for International Student Assessment (PISA) influencing global education agendas (Sellar & Lingard, 2013). The emphasis on standardised testing and academic attainment can sometimes create tensions with trauma-informed approaches.

The underlying philosophy of education - whether it is seen primarily as a means of academic credentialing or as a space for holistic development - also significantly affects the extent to which trauma-informed practices are embraced (Flook, et al., 2022; O’Toole & Simovska, 2022). A more humanistic view of education, which values wellbeing, citizenship and relational learning alongside academic achievement, provides a stronger foundation for sustaining trauma-informed approaches in the long term.

Beyond education policy, broader social and political conditions also shape the uptake and sustainability of trauma-informed practices. Geopolitical crises, such as war and displacement, increase the number of children who experience trauma, creating an urgent need for trauma-sensitive approaches in schools and in low-resource humanitarian contexts (Morrison et al., 2022; Yule, et al., 2013). These crises often involve physical destruction, repeated school closures, mobility restrictions, and other emergency conditions that have created a need for digital and remote learning modalities to safeguard children’s basic right to education. A challenge for future work in crises and emergency contexts will be developing technological solutions that still prioritise human connection and nurture relational care, dignity, and collective healing (Nath, 2025, UNICEF, 2025; UNESCO, 2025). The development of digital solutions will need to be guided by trauma-informed design principles and frameworks.

In an era marked by division, polarisation, and an erosion of trust, the world faces profound challenges - environmental destruction, social unrest, and deepening inequalities threaten the fabric of our shared humanity. In such a climate, championing a compassion-centred, trauma-informed approach in education is both urgent and challenging. Schools are not isolated from the turmoil but are embedded within it, grappling with political uncertainty, strained resources, and competing policy priorities. Yet, it is precisely in times of upheaval that trauma-informed practices become most vital, offering a foundation for hope, healing and humanity.

The EMBRACE Framework offers a potential contribution to future developments in trauma-informed education. Many of its components appear, explicitly or implicitly, across existing trauma-informed practices; however, EMBRACE’s distinctive value lies not in introducing entirely new elements but in how it synthesises, organises, and operationalises them into a coherent, compassion-centred structure that supports implementation across individual practice and organisational levels. It remains an emergent and evolving model. Whereas existing trauma-informed approaches often foreground individual components (relational pedagogies, nurture rooms, staff supervision) as standalone principles or parallel sets of best practice, EMBRACE deliberately integrates these elements through a unifying conceptual lens. By drawing on transdisciplinary perspectives and emphasising their interdependencies, the framework offers an integrative structure that can help schools move beyond fragmented or piecemeal adoption toward a more holistic, coherent, and system-wide orientation. There is work underway that uses the EMBRACE Framework in school contexts, and these initiatives are currently being explored and evaluated. Although detailed reporting is beyond the scope of this paper, an illustrative account of the framework’s application with practice-based vignettes is provided in McKernan and O’Toole (2026) and in Murphy and O’Toole (in press), where it is applied to the context of historical trauma in Irish education.

## Conclusion

In an era of mounting global crises and social upheaval, schools serve as vital spaces for healing and transformation. The EMBRACE Framework provides a roadmap for a more holistic, relational, and systemically integrated approach to trauma-informed education - one that moves beyond isolated interventions toward a sustained cultural shift in schools. By centring compassion, collaboration, and equity, educators and policymakers can cultivate environments where all students, particularly those who have experienced adversity, can flourish. This second wave of trauma-informed practice calls for a collective transdisciplinary commitment to reimagining schools not just as places of learning, but as ecosystems of care, connection, and hope.

## Declarations

### Supporting Documentation

There is no supplementary or supporting data related to this research.
